# Effect of Xylazine on Pharmacokinetics and Physiological Efficacy of Intravenous Carprofen in Castrated Goats Kids

**DOI:** 10.3390/ani13172700

**Published:** 2023-08-24

**Authors:** Kamil Uney, Murat Yuksel, Duygu Durna Corum, Devran Coskun, Erdinc Turk, Hasan Basri Dingil, Orhan Corum

**Affiliations:** 1 Department of Pharmacology and Toxicology, Faculty of Veterinary Medicine, University of Selcuk, Konya 42003, Türkiye; 2 Department of Obstetrics and Gynecology, Faculty of Veterinary Medicine, University of Hatay Mustafa Kemal, Hatay 31060, Türkiye; muyuksel@yahoo.com; 3 Department of Pharmacology and Toxicology, Faculty of Veterinary Medicine, University of Hatay Mustafa Kemal, Hatay 31060, Türkiye; ddurnacorum@gmail.com (D.D.C.); erdincturk48@gmail.com (E.T.); orhancorum46@hotmail.com (O.C.); 4 Department of Pharmacology and Toxicology, Faculty of Veterinary Medicine, University of Siirt, Siirt 56100, Türkiye; devrancoskun@gmail.com; 5 Department of Obstetrics and Gynecology, Institute of Health Sciences, University of Hatay Mustafa Kemal, Hatay 31060, Türkiye; vethekhbdingil@gmail.com

**Keywords:** castration, carprofen, cortisol, pharmacokinetics, xylazine

## Abstract

**Simple Summary:**

The castration of male goats is one of the most common livestock management procedures to reduce aggression and sexual behavior by lowering testosterone levels, prevent unwanted pregnancies, increase carcass composition and weight development, and reduce goaty smell. Carprofen can be used in the castration process of goats due to favorable pharmacological properties. This study investigated the effect of xylazine on the pharmacokinetics and physiological efficacy of intravenous carprofen in castrated male goat kids. Xylazine increased plasma concentration of carprofen and decreased clearance in castrated male goat kids. The increased cortisol concentration after castration was effectively reduced via the combined administration of carprofen and xylazine before castration.

**Abstract:**

Carprofen can be used in the castration process of male goats due to its low side effects, long elimination half-life, and long-term effect. However, no studies were found on the pharmacokinetics and physiological efficacy of carprofen when employed for castration in male goats. The aim of this study was to determine the effect of xylazine (0.05 mg/kg, intramuscular) on the pharmacokinetics and physiological efficacy following intravenous administration of carprofen (4 mg/kg, intravenous) in male goat kids castrated using the burdizzo method. Thirty male Kilis goat kids (5–6 months and 18–30 kg of body weight) were randomly assigned to five groups (*n* = 6) as follows: healthy control (HC), castration control (CAST), castration+carprofen (CAST+CRP), castration+xylazine (CAST+XYL), and castration+xylazine+carprofen (CAST+XYL+CRP). Plasma concentrations of carprofen were analyzed via a non-compartmental method. Physiological parameters including serum cortisol, scrotal temperature, rectal temperature, and scrotal circumference were determined. Xylazine caused a decrease in the volume of distribution and clearance and an increase in the area under the curve of carprofen in CAST+XYL+CRP group (*p* < 0.05). The mean cortisol concentrations in CAST+CRP and CAST+XYL remained lower compared to CAST (*p* < 0.05). The mean cortisol concentrations in CAST+XYL+CRP were lower than in CAST+CRP and CAST+XYL (*p* < 0.05). In addition, the effect of carprofen administration alone on reducing the initial cortisol response to castration was observed from 6 to 48 h, while in combination with xylazine, it was observed immediately up to 48 h. No treatment differences were observed in rectal temperature, scrotal temperature, and scrotal circumference (*p* > 0.05). Xylazine caused an increase in plasma concentration and a decrease in clearance of carprofen after co-administration. However, when the effect of the combined administration of carprofen with xylazine on cortisol is evaluated, their combined use in castration process may be beneficial.

## 1. Introduction

Goat is an important animal species with a strong socioeconomic impact in developing countries. The castration of male goats is one of the most common livestock management procedures in many countries. The benefits of castration include reducing aggression and sexual behavior by lowering testosterone levels, preventing unwanted pregnancies, increasing carcass composition, and reducing goaty smell [1,2]. Castration methods are generally divided into three major groups: physical, chemical, and hormonal. The most preferred of these methods are physical castration methods that include procedures such as surgical, burdizzo, latex band, and rubber ring [1,3]. These techniques allow the testicles to be surgically removed, irreversibly damaged, or atrophied by restricting blood flow [3]. All of these castration techniques cause varying degrees of physiological stress, inflammatory reactions, pain-related behaviors, and suppression of immune functions [4]. In order to reduce or suppress all these symptoms, local anesthetics, alpha-2 agonists, and nonsteroidal anti-inflammatory drugs (NSAIDs) are used [2].

NSAIDs are widely used in veterinary medicine due to their analgesic, antipyretic, and anti-inflammatory properties [5]. In small ruminants, NSAIDs are prescribed for painful and inflammatory conditions such as castration, mulesing, tail docking, lameness, mastitis, and pneumonia [6,7]. NSAIDs work by inhibiting the cyclooxygenase (COX) enzymes, which are responsible for the synthesis of prostaglandins from arachidonic acid [8]. COX-1 is constitutively expressed throughout the body to maintain normal physiological processes. COX-2 is generally inducibly expressed and triggered by growth factors, cytokines, and proinflammatory stimuli, while it is constitutively expressed in the kidney and plays an important role in renal homeostasis [9]. Therefore, NSAIDs with greater COX-2 selectivity are considered to have lower side effects on the gastrointestinal system. Carprofen belongs to the propionic acid class of NSAIDs. Carprofen has a single chiral molecule, and the commercially available formulation is a 50:50 racemic mixture of the S(+) and R(−) enantiomers [10]. NSAIDs having a COX-1/COX-2 ratio greater than 1 for inhibitory concentration (IC) 50 are thought to be more effective at suppressing COX-2. Carprofen has preferential inhibition on COX-2 with the COX-1/COX-2 ratio of >1 in cat (25.6), sheep (5.3–6.3), and dog (1.75) [11]. Carprofen has a low risk of gastric irritation and has a longer half-life than other NSAIDs such as meloxicam, ketoprofen, tolfenamic acid, and flunixin meglumine [6,7,12,13,14]. Carprofen is used for mastitis, respiratory diseases, musculoskeletal pain, surgery, or trauma pain and osteoarthritis in horses, cattle, dogs, and cats [15]. It is also used in an extra-label manner in painful and inflammatory conditions in goats [16].

Alpha-2 agonists have sedation, analgesia, and muscle relaxation effects. Alpha-2 adrenoceptors are located in the pre- and postsynaptic sites both centrally and peripherally. The sedative, analgesic, and muscle-relaxant effects of alpha-2 agonists result from their ability to reduce noradrenaline release mediated by central alpha-2 adrenoceptors [17]. Xylazine was the first alpha-2 agonist used in veterinary medicine and has been approved for use in many animal species [18]. In ruminants, xylazine is 10–20 times more potent than in other animal species [19].

Carprofen and xylazine are used to reduce pain caused by the castration in different animal species [20,21,22]. Xylazine has a short analgesic effect, resulting in inadequate postoperative analgesia [23]. The fact that NSAIDs provide long-term postoperative analgesia and have anti-inflammatory properties provides an advantage in use [24]. Although ketoprofen, flunixin meglumine, and carprofen have been used successfully in the castration of calves, it has been stated that the effects of ketoprofen and flunixin meglumine are short lived, and repeated administration is required [22]. Carprofen can be used for the castration of male goats due to its low side effects, long elimination half-life, and long-term effect. In addition, the simultaneous administration of xylazine and NSAIDs in castration showed a strong analgesic effect [25]. Based on the literature reviewed, no studies were found on the pharmacokinetics and efficacy of the castration of male goats. We hypothesized that xylazine would alter the plasma pharmacokinetics of carprofen with its physiological effects in the body and that the combination of xylazine and carprofen in castrated male goats would be much more effective in reducing plasma cortisol levels than single administrations. The aim of this study was to determine the effect of xylazine on the pharmacokinetics and efficacy of carprofen in castrated male goats.

## 2. Materials and Methods

### 2.1. Chemicals

The analytical standard for carprofen (97.0%) was bought from Sigma-Aldrich in St. Louis, MI, USA. Acetic acid, perchloric acid, Sodium acetate, and n-butyl acetate were obtained from Merck (Darmstadt, Germany). The analytical purity grade of methanol (VWR International, Fontenay-sous-Bois, France) was utilized for high-performance liquid chromatography (HPLC). The administration of carprofen (Rimadyl, 50 mg/mL, Injection Solution, Zoetis, Istanbul/Türkiye) and xylazine (Xylazinbio, 2%, Injection Solution, Bioveta, Ankara/Türkiye) with parenteral formulations was employed to provide analgesic effects to bucks.

### 2.2. Animals

This study was carried out on thirty male Kilis goat kids (5–6 months and 18–30 kg of body weight) that had not received any drugs since at least 1 month prior to the start of present research. The male goat kids were determined to be healthy via clinical examinations, complete blood count, and biochemistry analysis. The male goat kids were randomly divided into five equal groups of six animals each, and each group was taken to separate paddocks. They were moved into this compartment one week before the study to acclimate. The male goat kids were fed with the ration suitable for their age and developmental stages during the study period, and they had ad libitum access to water and hay. All study procedures were approved (2023/02-01) by the Local Ethics Committee for Animal Research Studies at Hatay Mustafa Kemal University, (Hatay, Türkiye) and carried out in accordance with the European Directive (2010/63/EU).

### 2.3. Group Assignment and Randomization Procedure

Thirty male goat kids (5–6 months and 18–30 kg of body weight) were randomly assigned to five groups (n = 6) as follows: healthy control (no castration, HC), castration control (CAST), castration+carprofen (CAST+CRP), castration+xylazine (CAST+XYL), castration+xylazine+carprofen (CAST+XYL+CRP). Groups were established by drawing closed papers with numbers ranging from 1 to 30 at random, and goats kids were then numbered using oil paint. They were weighed 12 h before castration to calculate individual dosages.

### 2.4. Drug Administration and Castration Prosedure

Goat kids assigned to CAST+CRP and CAST+XYL+CRP were given intravenous (IV) carprofen at a dose of 4 mg/kg. Goat kids assigned to CAST+XYL and CAST+XYL+CRP were given intramuscular (IM) xylazine at a dose of 0.05 mg/kg. Goat kids in HC and CAST were intravenously administered 0.9% sterile sodium chloride (Polifarma, Istanbul/Türkiye) in volume equivalent to 4 mg/kg carprofen. Xylazine was administered 10 min before carprofen, and carprofen was administered immediately prior to the castration. IV and IM administration were performed on the left jugular vein and between the semitendinosus and the semimembranosus muscles, respectively. Goats kids were castrated using the Burdizzo (emasculatome) method. Castration was carried out at 3 min intervals. To minimize variation, all castrations were performed by a single experienced operator.

### 2.5. Blood Sample Collection

Blood samples were collected from the right jugular vein via a catheter (22 G, 0.9 × 25 mm, Bıcakcılar Medical Devices Industry and Trade Co., Istanbul, Türkiye) in the first 24 h and by venipucture (21 G, 0.8 × 35 mm, Genject Health Products, Ankara, Türkiye) at other sampling times. To determine the pharmacokinetics of carprofen, blood samples (2 mL) from CAST+CRP and CAST+XYL+CRP were collected into tubes containing heparin at 0 (before administration of carprofen), 0.083, 0.25, 0.5, 1, 2, 4, 6, 8, 10, 12, 24, 48, 96, 144, 192, 240, and 288 h after drug administration. For cortisol analysis, blood samples (2 mL) from all groups were collected into gel-containing tubes at −0.17 (before administration of xylazine), 0 (prior to castration), 0.5, 1, 6, 12, 24, 48, 96, 144, 192, 240, and 288 h after castration. All blood samples were centrifuged at 4000× *g* for 10 min, and the separated plasma and serum were stored at −80 °C until analysis.

### 2.6. Analysis of Carprofen

Carprofen concentrations in goat plasma were assayed using the HPLC by modifying the previous published method [26,27]. An amount of 300 µL of acetate buffer (1 M, pH: 2.8) was added to 200 µL of plasma sample and vortexed for 45 s, and then 4 mL of n-butyl acetate was added to this mixture. They were stirred for 40 s in the vortex, then centrifuged at 12,500× *g* for 15 min. The organic phase was then transferred to another tube and evaporated at 40 °C under nitrogen. The residual substance was combined with a volume of 200 μL of the mobile phase and afterwards transferred into vials designed for use with an auto-sampler. A volume of 20 μL of supernatant was introduced into a Gemini^TM^ C18 column (250 × 4.6 mm; internal diameter, 5 μm, GL Sciences, Japan) maintained at a temperature of 30 °C. The mobile phase consisted of 70% methanol and 30% aqueous solution (containing 50 μL of 0.2% perchloric acid) at a flow rate of 1 mL/min. The HPLC system (Shimadzu, Tokyo, Japan) consisted of an auto-sampler (SIL 20A), a degasser (DGU-20A), a column oven (CTO-10A), a pump (LC-20AT), and an UV–VIS detector (SPD-20A). Carprofen was detected at a wavelength of 254 nm.

The validation of the chromatographic method was conducted in accordance with the guidelines provided by the European Medicines Agency [28]. The carprofen stock solution was prepared by dissolving in methanol to achieve a concentration of 1 mg/mL. By diluting the stock solution, working standard solutions of varying concentrations (0.02, 0.04, 0.1, 0.2, 0.4, 1, 2, 4, 10, 20, 40, and 100 μg/mL) were obtained. The selectivity of method was evaluated by extracting blank plasma samples from individual animals for interference from plasma. Calibration standards (0.02, 0.04, 0.1, 0.2, 0.4, 1, 2, 4, 10, 20, 40, and 100 μg/mL) and quality control samples were prepared by adding working standard solutions of carprofen into blank goat plasma. Carprofen calibration curve was linear (R^2^ > 0.9992) over the range of 0.02–100 μg/mL. The quality control samples (0.04, 4, and 40 μg/mL) were analyzed in 5 replicates within 5 days to assess recovery, precision, and accuracy. The recovery of carprofen was ≥92%. The lower limit of quantification was 0.02 μg/mL for carprofen in goat plasma with the bias of ±15% and the coefficient of variation <20%. The intra-day and inter-day coefficients of variation for precision were ≤6.4% and ≤7.2%, respectively. The intra-day and inter-day bias for accuracy were ±7.6% and ±8.4%, respectively.

### 2.7. Pharmacokinetic Analysis

Using the WinNonlin 6.1.0.173 software program (Pharsight Corporation, Scientific Consulting Inc., Cary, NC, USA), plasma concentrations of carprofen in each goats were calculated via noncompartmental analysis. After IV administration, total clearance (Cl_T_), volume of distribution at steady state (V_dss_), terminal elimination half-life (t_1/2λz_)_,_ mean residence time (MRT), area under the concentration versus time curve (AUC), and AUC extrapolated from tlast to ∞ in % of the total AUC (AUC_extrap_ %) were determined. The peak plasma concentration (C_0.083h_) was directly obtained from first sampling (0.083 h) data of concentration–time curves. The body extraction ratio (E_body_) for carprofen was calculated using C_lT_/Q_C_, and Q_C_ (mL/kg/min) was the cardiac output calculated according to the allometric equation with 180 × body weight (in kg)^−0.19^ [29].

### 2.8. Physiological Parameters

Cortisol analysis from serum samples was performed in an ELISA reader (MWGt Lambda Scan 200, Bio-Tek Instruments, Winooski, VT, USA) as specified by the supplier using commercial ELISA kits (BT Lab., Shanghai, China) specific to the goat. The standard curve range and sensitivity of the test were 1–400 ng/mL and 0.52 ng/mL. The correlation coefficient of the standard curve range was >0.9987. Quality control samples of low (1 ng/mL), medium (10 ng/mL), and high (100 ng/mL) levels of cortisol were used to determine the precision and accuracy, and 3 different analyses were performed on 3 different days for each level. The calculated coefficient of variation for intra- assay variability was <8.0%, and the inter-assay variability was <9.6%.

Scrotal temperature (Infrared camera, UTi712s, Unit), rectal temperature (Livestock thermometer, HaiTun) and scrotal circumference (Reliabull Scrotal Tape, Lane Manufacturing) were measured at −0.17 (before administration of xylazine), 0 (prior to castration), 0.5, 1, 6, 12, 24, 48, 96, 144, 192, 240 and 288 h after castration. The evaluations were performed by the same person to minimize variation.

### 2.9. Statistical Analysis

The statistical analysis was performed utilizing the SPSS 22.0 software program (IBM Corp., Armonk, NY, USA). The statistical significance level was set at *p* < 0.05. The normality of the data distribution was assessed using the Shapiro–Wilk test, while the homogeneity of the variance was assessed using the Levene test. Pharmacokinetic parameters were presented as geometric mean (min-max) and analyzed using an independent *t*-test. The comparison of group means in physiological parameters was analyzed using one-way analysis of variance (ANOVA) and post hoc Tukey tests. Effects of time in all physiological parameters were conducted using the ANOVA for repeated measurements.

## 3. Results

### 3.1. Pharmacokinetic Parameters

**Figure 1:**
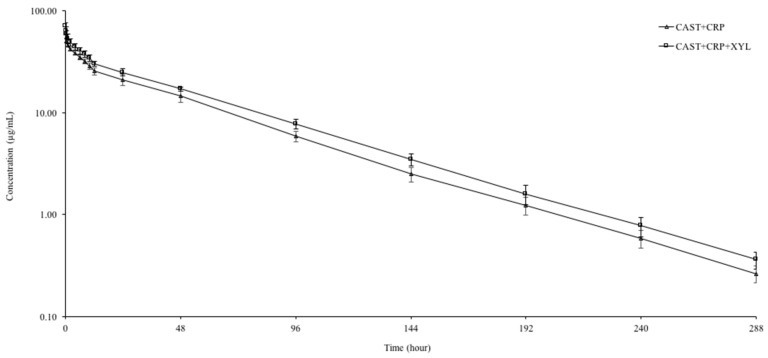
Semi-logarithmic plasma concentration–time curves after intravenous administration of carprofen (CRP, 4 mg/kg) alone (CAST+CRP) and co-administered (CAST+XYL+CRP) with xylazine (0.05 mg/kg, IM) in castrated male goat kids (mean ± SD, n = 6).

The semi–logarithmic plasma concentration–time curves and pharmacokinetic parameters after IV administration of carprofen alone (CAST+CRP) and co-administered (CAST+XYL+CRP) with xylazine in castrated goat kids are presented in Figure 1 and Table 1, respectively. Carprofen was detected in plasma up to 288 h in CAST+CRP and CAST+XYL+CRP groups. Xylazine raised carprofen plasma concentrations from 60.14 to 69.82 µg/mL at the first sampling (0.083 h) and from 0.26 to 0.35 µg/mL at the last sampling (288 h). The t_1/2λz_, Cl_T_, V_dss_, and AUC_0–∞_ values after carprofen alone administration were 41.59 h, 2.08 mL/h/kg, 116.49 mL/kg, and 1925.48 h*g/mL, respectively. Xylazine administration decreased Cl_T_ and V_dss_ while increased AUC_0–∞_ (*p* < 0.05). The administration of xylazine did not change the t_1/2λz_ and MRT_0–∞_ of carprofen. E_body_ values of carprofen after IV administration were 0.0087 (0.0062–0.0104) and 0.0081 (0.0069–0.0092) after alone and simultaneous administration with xylazine, respectively. The estimated percent AUC extrapolated values were less than 20% for both groups.

### 3.2. Physiological Parameters

**Figure 2:**
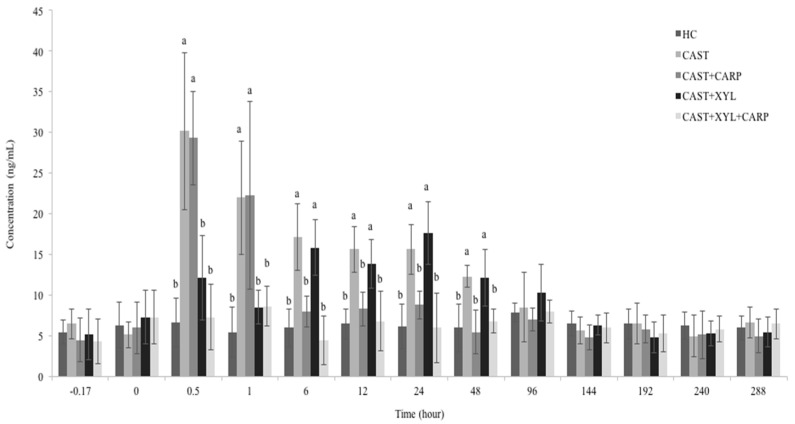
Effect of different treatments and castration on plasma cortisol concentrations in male goat kids (mean ± SD). ^ab^: Different superscripts indicate statistical difference (*p* < 0.05). (HC: healthy control, CAST: Castration, CRP: Carprofen, XYL: Xylazine).

To minimize the effect of blood draws on cortisol levels, goats were accustomed to handling and blood draws were performed with little or no restriction. The effect of different treatments and castration on physiological parameters in male goat kids are presented in Table 2. Mean cortisol concentrations differed significantly between treatment groups (*p* < 0.05). The mean cortisol concentrations in CAST+CRP and CAST+XYL remained lower compared to CAST (*p* < 0.05). The mean cortisol concentrations in CAST+XYL+CRP were lower than in CAST+CRP and CAST+XYL in which these drugs were administered alone (*p* < 0.05). Cortisol concentrations were higher from 0.5 to 48 h in CAST compared to HC (Figure 2). Compared to CAST, cortisol concentrations were significantly lower at 0.5 and 1 h in CAST+XYL and at 6, 12, 24 and 48 h in the CAST+CRP (Figure 2). In CAST+XYL+CRP, cortisol concentrations were lower at 0.5, 1, 6, 12, 24 and 48 h compared to CAST (Figure 2). There was no difference in cortisol level at other times. Mean rectal temperature, scrotal temperature and scrotal circumference values were 39.02–39.15 °C, 35.87–36.15 °C and 18.81–19.32 cm, respectively. There was no difference in these values between the treatment groups (*p* > 0.05).

## 4. Discussion

In this study, the pharmacokinetics and physiological efficacy of carprofen after alone and co-administered with xylazine in castrated male goat kids by the burdizzo method were revealed for the first time. The burdizzo technique is a bloodless castration procedure that may be performed on animals of any age and that is less painful than other physical castration procedures [1]. Carprofen and xylazine are not approved for use in goats in our country and are used extra-label. Carprofen is used extra-label at doses of 2–4 mg/kg in goats [16,30]. Carprofen was well tolerated after single and repeated intravenous administration at a dose of 4 mg/kg to sheep [31,32]. It was reported that IV and extravascular (IM, oral, subcutaneous) t_1/2λz_ of carprofen in sheep were similar and peak plasma concentration was reached in 2.06–11.73 h after extravascular administration [27]. IV administration may be preferred for rapid onset of effect without absorption effect. IV and IM administration of xylazine is recommended [33] and has been successfully used in goats at a dose of 0.05 mg/kg [23,34]. In this study, the castration method, drug doses and administration routes were preferred for the above reasons.

The _t1/2λz_ of carprofen in CAST+CRP was found to be 41.59 h, which is consistent with recent findings in sheep (43.36 h) [27] and calves (43.4 h) [35]. Notably, this _t1/2λz_ is longer than that reported for horse, cat, dog, and cows (8.00–30.7 h) [36,37,38,39]. The V_dss_ of carprofen in CAST+CRP was determined to be 116.49 mL/kg. This value is similar to the V_dss_ values previously reported for sheep (92.7–121.36 mL/kg) [27,40], dog (140 mL/kg) [37], cat (140 mL/kg) [39], and calves (154.7 mL/kg) [35]. The Cl_T_ in CAST+CRP was 2.08 mL/h/kg, which was similar to that reported for sheep, cows and calves (1.98–2.45 mL/h/kg) [27,35,36], and lower than that reported for cat and dog (7.10–14.87 mL/h/kg) [39,41].

In castrated male goat, xylazine administration decreased the V_dss_ of carprofen from 116.49 to 99.45 mL/kg. Similarly, xylazine decreased the V_dss_ of meloxicam in castrated goats and ketoprofen in mice [7,42]. The volume of distribution is influenced by body composition, ionization state, and binding of drugs to plasma proteins [43]. The binding ratio of carprofen to plasma proteins is not known in goats, but it is highly bound (>99%) in cattle, dogs and horses. Carprofen is weakly acidic (pKa: 4.3) and ionized at plasma pH [12]. Carprofen may have a low V_dss_ because it binds to plasma proteins strongly and becomes ionized at the pH of plasma. Xylazine administration to goats causes a decrease in heart rate, respiratory rate and hemoglobin levels. In addition, it causes changes in blood pH, bicarbonate, oxygen and carbon dioxide levels [19,23]. These effects may affect the blood supply of the tissue and ultimately its volume of distribution [44]. Xylazine is not extensively bound to plasma proteins [17]. Therefore, the decrease in V_dss_ of carprofen may be due to the hemodynamic effects of xylazine.

Xylazine administration reduced the Cl_T_ of carprofen from 2.08 to 1.69 mL/h/kg in castrated male goats. Xylazine also reduced the Cl_T_ of meloxicam in castrated goats and ketoprofen in mice [7,42]. E_body_ of carprofen after alone and co-administered with xylazine in castrated goat kids was 0.0087 and 0.0081, respectively. Since E_body_ is stated as 0.05 for low, 0.15 for medium, and 0.35 for high [24], these values show that this drug has a low extraction rate after alone, and simultaneous administration with xylazine in castrated goat. Carprofen undergoes variable rates of phase I and phase II reactions in mammals, with the majority of the drug being eliminated as conjugate metabolites in urine (8–70%) and bile (35–75%) [10,45]. In humans, less than 5% of the dose is excreted in the urine as intact carprofen [45]. Xylazine is extensively metabolized by phase I and phase II reactions in rats and excreted via the urine (70%) and feces (30%) [33]. After xylazine administration to goats, physiological changes started at 5 min and sedation occurred for approximately 60 min [23,46]. In addition, it has been stated that xylazine is detected up to 2.5–96 h in various animal species in the analyses carried out with various analysis methods [47,48,49]. It is reported that xylazine causes dose-related cardiovascular depression and reduces the heart rate by 30% [19]. As a result, it reduces blood flow in organs such as the brain, kidney, and liver [50,51]. The decrease in Cl_T_ of carprofen observed in this study may have been caused by decreased blood flow to elimination organs such as the liver and kidney as a result of the cardiovascular effects of xylazine, or by a reduction in carprofen biotransformation.

Xylazine administration increased the C_0.083 h_ (from 60.14 to 69.82 µg/mL) and AUC_0–∞_ (from 1925.48 to 2361.32 h*µg/mL) of carprofen in castrated goat kids. Xylazine raised the AUC_0–∞_ and peak concentration values of meloxicam in castrated goats and ketoprofen in mice, which was similar to our findings [7,42]. In this study, xylazine was administered 10 min before carprofen. It has been reported that physiological effects begin 5 min after xylazine administration to goats [23,46]. Xylazine administration increased the plasma concentration of carprofen from 0.26 to 0.35 µg/mL at the last sampling (288 h). The increase in AUC_0–∞_ and C_max_ values in castrated goat kids may be due to the change in the plasma concentration time curve of carprofen due to the physiological effects of xylazine.

The mean cortisol concentrations in CAST+CRP and CAST+XYL decreased compared to CAST. The mean cortisol concentrations in CAST+XYL+CRP were lower than in CAST+CRP and CAST+XYL. Compared to CAST, cortisol concentrations were significantly lower at 0.5 and 1 h in CAST+XYL and at 6, 12, 24, and 48 h in the CAST+CRP. It was stated that cortisol concentration increased up to 72 h after castration via the burdizzo method [4,22]. Acute pain causes the secretion of cortisol by activating the hypothalamic–pituitary–adrenal axis. Since cortisol suppresses immune-inflammatory reactions, its secretion for a long time can make the animal susceptible to infections [22]. Therefore, the pain caused by the castration procedure should be minimized. After administering xylazine to goats, analgesic activity began 9 min later and lasted 134 min [23]. The reason why xylazine reduces cortisol level up to 1 h may be due to its short analgesic effect. The effect of carprofen on cortisol was seen from 6 h in castrated goat kids. Similarly, the effect of carprofen on cortisol was seen after 6–12 h in castrated bulls [52]. It has been reported that the inhibitory effect of tolfenamic acid (another NSAID) on prostaglandin E_2_ appeared after 4 h in the carrageenan-induced inflammation model in dogs. Also, tolfenamic acid reached peak concentrations in plasma and exudate at 0.3–1.3 h and 5.14–6.75 h, respectively [53]. The reason for the late onset of the effect of carprofen on cortisol may be due to its lateness in reaching the effective concentration in the pain area. CAST+XYL+CRP may have been more effective on cortisol because of the rapid onset of the analgesic effect of xylazine and the long-term analgesia of carprofen.

In this study, no difference was observed between the groups in mean rectal temperature, scrotal temperature, and scrotal circumference. It has been stated that these values do not change in many studies performed in castrated calves [54,55,56]. The effect of xylazine on rectal temperature in goats is variable, and it had no effect on rectal temperature in one study [46], but reduced it in another study [23]. In this study, the scrotal circumference was measured to obtain information about inflammation. The reason why no difference was observed in scrotal circumference may be that both cortisol and carprofen have anti-inflammatory effects. In addition, inflammation after castration may be age related. After castration, inflammation occurred in lambs 1–6 weeks old, but not in 6-month-old sheep [22]. Another reason for the lack of difference in scrotal circumference may be that 5–6-month-old goat kids were used in this study.

The limitation of this study is the inability to determine the plasma protein binding ratio and metabolism of carprofen after single and simultaneous administration with xylazine in castrated goats. In addition, the lack of determination of the effect of different treatments on pain score, inflammatory parameters, and behavioral parameters can be considered as a limitation of this study.

In this study, no analgesia treatment was administration to the castration group. This situation can be seen as problematic from an ethical point of view. However, in many previous studies on this subject, analgesics were not administered to the castration group [4,22,52,55]. In addition, survey studies reported that only 20% of veterinarians used analgesia in castration [3]. Whatever procedures are performed, the castration process causes pain and stress. However, analgesic administration in castration may change the pain and stress response. Therefore, we did not apply analgesia treatment to the castration group in order to determine the changes in cortisol and physiological parameters in goat castration and the effects of single and combined administration of carprofen and xylazine on this change.

## 5. Conclusions

In conclusion, xylazine increased plasma concentration of carprofen and decreased clearance in castrated male goat kids. Castration and carprofen and xylazine administration have no effect on rectal temperature, scrotal temperature, and scrotal circumference. The effect of carprofen administration alone on reducing the initial cortisol response to castration was observed from 6 to 48 h, while in combination with xylazine, it was observed from the first moment to 48 h. Therefore, increased cortisol concentration after castration was effectively reduced by the combined administration of carprofen and xylazine before castration.

## Figures and Tables

**Table 1:** Pharmacokinetic parameters obtained after intravenous administration of carprofen (4 mg/kg) alone (CAST+CRP) and co-administered (CAST+XYL+CRP) with xylazine (0.05 mg/kg, IM) in castrated male goat kids.

Parameters	CAST+CRP	CAST+CRP+XYL
t_1/2λz_ (h)	41.59 (39.48–44.95)	43.14 (40.90–46.01)
AUC_0–last_ (h*µg/mL)	1909.58 (1686.20–2143.96)	2338.87 (2180.57–2472.49) *
AUC_0–∞_ (h*µg/mL)	1925.48 (1699.32–2160.26)	2361.32 (2198.96–2498.05) *
AUC_extrap_ (%)	0.81 (0.65–1.19)	0.94 (0.70–1.25)
MRT_0–∞_ (h)	56.07 (53.41–58.49)	59.00 (54.86–65.10)
Cl_T_ (mL/h/kg)	2.08 (1.89–2.35)	1.69 (1.60–1.74) *
V_dss_ (mL/kg)	116.49 (105.83–129.82)	99.95 (94.43–106.71) *
C_0.083h_ (µg/mL)	60.14 (56.54–65.34)	69.82 (63.08–76.64) *

* Value is statistically different than that in the administration of carprofen (*p* ≤ 0.05). Data were presented as geometric mean (min–max). t_1/2λz_, terminal elimination half-life; AUC, area under the plasma concentration-time curve; AUC_extrap_ %, area under the plasma concentration-time curve extrapolated from tlast to ∞ in % of the total AUC; MRT, mean residence time; Cl_T_, total clearance; V_dss_, volume of distribution at steady state; C_0.083h_, plasma concentration at time 0.083 h.

**Table 2:** Effect of different treatments and castration on physiological parameters in male goat kids (mean ± SD).

			Treatment				
Parameters	Hour *p*-Value	Treatment *p*-Value	HC	CAST	CAST+CRP	CAST+XYL	CAST+CRP+XYL
Serum cortisol (ng/mL)	0.0000000	0.0000001	6.29 ± 0.69 ^c^	12.08 ± 2.41 ^a^	9.28 ± 2.76 ^b^	9.62 ± 1.11 ^b^	6.42 ± 0.98 ^c^
Rectal temperature (°C)	0.1039768	0.755362	39.15 ± 0.27	39.02 ± 0.45	39.06 ± 0.53	39.05 ± 0.37	39.13 ± 0.41
Scrotal temperature (°C)	0.5756055	0.2895756	36.15 ± 0.25	36.12 ± 0.42	35.87 ± 0.17	35.89 ± 0.32	36.14 ± 0.48
Scrotal sircumference (cm)	0.6914008	0.9996859	18.81 ± 0.31	19.28 ± 0.37	19.09 ± 0.39	19.32 ± 0.66	18.97 ± 0.90

^abc^: Different superscripts indicate statistical difference (*p* < 0.05). HC: healthy control, CAST: Castration, CRP: Carprofen, XYL: Xylazine.

## Data Availability

The data presented in this study are available on request from the corresponding author.

## References

[1] Yami A., Merkel R.C., Dawson L. (2004). Castration of Sheep and Goats.

[2] Stafford K.J., Mellor D.J. (2005). The welfare significance of the castration of cattle: A review. N. Z. Vet. J..

[3] Coetzee J.F. (2013). Assessment and management of pain associated with castration in cattle. Food Anim. Pract..

[4] Pang W.Y., Earley B., Murray M., Sweeney T., Gath V., Crowe M.A. (2011). Banding or burdizzo castration and carprofen administration on peripheral leukocyte inflammatory cytokine transcripts. Res. Vet. Sci..

[5] Fitzpatrick J., Scott M., Nolan A. (2006). Assessment of pain and welfare in sheep. Small Rumin. Res..

[6] Corum O., Corum D.D., Er A., Yildiz R., Uney K. (2018). Pharmacokinetics and bioavailability of tolfenamic acid in sheep. J. Vet. Pharmacol. Ther..

[7] Tekeli I.O., Turk E., Durna Corum D., Corum O., Uney K. (2020). Effect of castration procedure on the pharmacokinetics of meloxicam in goat kids. J. Vet. Pharmacol. Ther..

[8] Mitchell M.A. (2005). Carprofen. Semin. Avian Exot. Pet Med..

[9] Hawkey C.J. (2001). COX-1 and COX-2 inhibitors. Best Pract. Res. Clin. Gastroenterol..

[10] Uney K., Durna Corum D., Terzi E., Corum O. (2021). Pharmacokinetics and bioavailability of carprofen in rainbow trout (Oncorhynchus mykiss) broodstock. Pharmaceutics.

[11] Hunter T.S., Robison C., Gerbino P.P. (2015). Emerging evidence in NSAID pharmacology: Important considerations for product selection. Am. J. Manag. Care.

[12] CVMP (1995). Carprofen Summary Report (1). European Agency for the Evaluation of Medicinal Products, EMEA/MRL/042/95-FINAL. https://www.ema.europa.eu/en/documents/mrl-report/carprofen-summary-report-1-committee-veterinary-medicinal-products_en.pdf.

[13] Turk E., Tekeli I.O., Durna Corum D., Corum O., Altinok Yipel F., Ilhan A., Emiroglu S.B., Uguz H., Uney K. (2021). Pharmacokinetics of tolfenamic acid in goats after different administration routes. J. Vet. Pharmacol. Ther..

[14] Welsh E.M., McKellar Q.A., Nolan A.M. (1993). The pharmacokinetics of flunixin meglumine in the sheep. J. Vet. Pharmacol. Ther..

[15] Papich M.G. (2016). Saunders Handbook of Veterinary Drugs: Small and Large Animal.

[16] Matthews J. (2016). Diseases of the Goat.

[17] Pawson P.S., Maddison J.E., Page S., Church D.B. (2008). Small Animal Clinical Pharmacology.

[18] Paddleford R.P., Harvey R.C. (1999). Alpha2 agonists and antagonists. Vet. Clin. N. Am.-Small Anim. Pract..

[19] Kastner S.B.R. (2006). A2-agonists in sheep: A review. Vet. Anaesth. Analg..

[20] Searle D., Dart A.J., Dart C.M., Hodgson D.R. (1999). Equine castration: Review of anatomy, approaches, techniques and complications in normal, cryptorchid and monorchid horses. Aust. Vet. J..

[21] Steiner B., Kamm A., Bettschart-Wolfensberger R. (2003). Influences of carprofen and the experience of the surgeon on post-castration pain in lambs and young sheep. Vet. Anaesth. Analg..

[22] Stilwell G., Lima M.S., Broom D.M. (2008). Effects of nonsteroidal anti-inflammatory drugs on long-term pain in calves castrated by use of an external clamping technique following epidural anesthesia. Am. J. Vet. Res..

[23] Kinjavdekar P., Amarpal G.S., Aithal H.P., Pawde A.M. (2000). Physiologic and biochemical effects of subarachnoidally administered xylazine and medetomidine in goats. Small Rumin. Res..

[24] Galatos A.D. (2011). Anesthesia and analgesia in sheep and goats. Vet. Clin. N. Am. Food Anim. Pract..

[25] González L.A., Schwartzkopf-Genswein K.S., Caulkett N.A., Janzen E., McAllister T.A., Fierheller E., Schaefer A.L., Haley D.B., Stookey J.M., Hendrick S. (2010). Pain miti-gation after band castration of beef calves and its effects on performance, behavior, escherichia coli, and salivary cortisol. Anim. Sci. J..

[26] Turk E., Tekeli I.O., Corum O., Durna Corum D., Kirgiz F.C., Cetin G., Atessahin D.A., Uney K. (2021). Pharmacokinetics of meloxicam, carprofen, and tolfenamic acid after intramuscular and oral administration in Japanese quails (Coturnix coturnix japonica). J. Vet. Pharmacol. Ther..

[27] Coskun D., Corum O., Durna Corum D., Uney K., Elmas M. (2022). Pharmacokinetics and bioavailability of carprofen in sheep. J. Vet. Pharmacol. Ther..

[28] EMA (2011). 2011. Guideline on Bioanalytical Method Validation. Committee for Medicinal Products for Human Use (CHMP), European Medecines Agency (EMA). https://www.ema.europa.eu/documents/scientific-guideline/guideline-bioanalytical-method-validation_en.pdf.

[29] Toutain P.L., Bousquet-Melou A. (2004). Plasma clearance. J. Vet. Pharmacol. Ther..

[30] Kutter A.P., Kästner S.B.R., Bettschart-Wolfensberger R., Huhtinen M. (2006). Cardiopulmonary effects of dexmedetomidine in goats and sheep anaesthetised with sevoflurane. Vet. Rec..

[31] Corum O., Coskun D., Durna Corum D., Ider M., Yildiz R., Ok M., Uney K. (2022). Pharmacokinetics of carprofen following single and repeated intravenous administrations of different doses in sheep. J. Vet. Pharmacol. Ther..

[32] Durna Corum D., Yıldız R. (2020). Effect of multiple-dose administration of carprofen on hematological and biochemical parameters in sheep. Eurasian J. Vet. Sci..

[33] (1999). CVMP. https://www.ema.europa.eu/en/documents/mrl-report/xylazine-hydrochloride-summary-report-1-committee-veterinary-medicinal-products_en.pdf.

[34] Abouelfetouh M.M., Liu L., Salah E., Sun R., Nan S., Ding M., Ding Y. (2021). The Effect of Xylazine Premedication on the Dose and Quality of Anesthesia Induction with Alfaxalone in Goats. Animals.

[35] Delatour P., Foot R., Foster A.P., Baggo D., Lees P. (1996). Pharmacodynamics and chiralpharmacokinetics of carprofen in calves. Br. Vet. J..

[36] Lohuis J.A.C.M., Van Werven T., Brand A., Van Miert A.S.J.P.A.M., Rohde E., Ludwig B., Heizmann P., Rehm W.F. (1991). Pharmacodynamics and pharmacokinetics of carprofen, a non-steroidal anti-inflammatory drug, in healthy cows and cows with Escherichia coli endotoxin-induced mastitis. J. Vet. Pharmacol. Ther..

[37] McKellar Q.A., Pearson T., Bogan J.A., Gaibraith E.A., Lees P., Ludwig B., Tiberghien M.P. (1990). Pharmacokinetics, tolerance and serum thromboxane inhibition of carprofen in the dog. J. Small Anim. Pract..

[38] McKellar Q.A., Bogan J.A., von Fellenberg R.L., Ludwig B., Cawley G.D. (1991). Pharmacokinetic, biochemical and tolerance studies on carprofen in the horse. Equine Vet. J..

[39] Parton K., Balmer T.V., Boyle J., Whittem T., MacHon R. (2000). The pharmacokinetics and effects of intravenously administered carprofen and salicylate on gastrointestinal mucosa and selected biochemical measurements in healthy cats. J. Vet. Pharmacol. Ther..

[40] Welsh E.M., Baxter P., Nolan A.M. (1992). Pharmacokinetics of carprofen administered intravenously to sheep. Res. Vet. Sci..

[41] Messenger K.M., Wofford J.A., Papich M.G. (2016). Carprofen pharmacokinetics in plasma and in control and inflamed canine tissue fluid using in vivo ultrafiltration. J. Vet. Pharmacol. Ther..

[42] Khalil K.A., Mousa Y.J., Alzubaidy M.H. (2022). Pharmacokinetic Criteria of Ketoprofen and its Cyclooxygenase-2 Inhibition in Mice: Influence of Xylazine Administration. Maced. Vet. Rev..

[43] Sakai J.B., Sakai J.B. (2009). Pharmacokinetics: The absorption, distribution, and excretion of drugs. Practical Pharmacology for the Pharmacy Technician.

[44] Ernstmeyer K., Christman E. (2023). Nursing Pharmacology.

[45] Rubio F., Seawall S., Pocelinko R., DeBarbieri B., Benz W., Berger L., Morgan L., Pao L., Williams T.H., Koechlin B. (1980). Metabolism of carprofen, a nonsteroidal anti-inflammatory agent, in rats, dogs, and humans. J. Pharm. Sci..

[46] Nahvi A., Molaei M.M., Samimi A.S., Azari O., Mashayekhi H., Ebrahimzadeh F. (2022). Evaluation of the sedative and physiological effects of xylazine, detomidine, medetomidine and dexmedetomidine in goats. Vet. Med. Sci..

[47] Santonastaso A., Hardy J., Cohen N., Fajt V. (2014). Pharmacokinetics and pharmacodynamics of xylazine administered by the intravenous or intra-osseous route in adult horses. J Vet. Pharmacol. Ther..

[48] Garcia-villar R., Toutain P.L., Alvinerie M., Ruckebusch Y. (1981). The pharmacokinetics of xylazine hydrochloride: An interspecific study. J. Vet. Pharmacol. Ther..

[49] Knych H.K., Stanleya S.D., McKemiea D.S., Arthurc R.M., Kass P.G. (2017). Pharmacokinetic and pharmacodynamics of xylazine administered to exercised thoroughbred horses. Drug Test. Anal..

[50] Symonds H.W. (1976). The effect of xylazine upon hepatic glucose pro- duction and blood flow rate in the lactating dairy cow. Vet. Rec..

[51] Talke P.O., Traber D.L., Richardson C.A., Harper D.D., Traber L.D. (2000). The effect of α_2_ agonist-induced sedation and its reversal with an α_2_ antagonist on organ blood flow in sheep. Anaesth. Analg..

[52] Pang W.Y., Earley B., Sweeney T., Crowe M.A. (2006). Effect of carprofen administration during banding or burdizzo castration of bulls on plasma cortisol, in vitro interferon-γ production, acute-phase proteins, feed intake, and growth. J Anim Sci..

[53] McKellar Q.A., Lees P., Gettinby G. (1994). Pharmacodynamics of tolfenamic acid in dogs. Evaluation of dose response relationships. Eur. J. Pharmacol..

[54] Meléndez D.M., Marti S., Pajor E.A., Moya D., Gellatly D., Janzen E.D., Schwartzkopf-Genswein K.S. (2018). Effect of a single dose of meloxicam prior to band or knife castration in 1-wk-old beef calves: I. Acute pain. J. Anim. Sci..

[55] Meléndez D.M., Marti S., Pajor E.A., Sidhu P.K., Gellatly D., Moya D., Schwartzkopf-Genswein K.S. (2018). Effect of meloxicam and lidocaine administered alone or in combination on indicators of pain and distress during and after knife castration in weaned beef calves. PLoS ONE.

[56] Meléndez D.M., Marti S., Pajor E.A., Moya D., Gellatly D., Janzen E.D., Schwartzkopf-Genswein K.S. (2018). Effect of subcutaneous meloxicam on indicators of acute pain and distress after castration and branding in 2-mo-old beef calves. J. Anim. Sci..

